# Identification of genes involved in male sterility in wheat (*Triticum aestivum* L.) which could be used in a genic hybrid breeding system

**DOI:** 10.1002/pld3.201

**Published:** 2020-03-10

**Authors:** Matthew J. Milner, Melanie Craze, Sarah Bowden, Ruth Bates, Emma J. Wallington, Anthony Keeling

**Affiliations:** ^1^ The John Bingham Laboratory NIAB Cambridge UK; ^2^ Elsoms Developments Ltd/Elsoms Seeds Ltd Spalding UK

**Keywords:** callose synthase, gene editing, hybrid wheat, pollen fertility, ruptured pollen grain‐1

## Abstract

Wheat is grown on more land than any other crop in the world. Current estimates suggest that yields will have to increase sixty percent by 2050 to meet the demand of an ever‐increasing human population; however, recent wheat yield gains have lagged behind other major crops such as rice and maize. One of the reasons suggested for the lag in yield potential is the lack of a robust hybrid system to harness the potential yield gains associated with heterosis, also known as hybrid vigor. Here, we set out to identify candidate genes for a genic hybrid system in wheat and characterize their function in wheat using RNASeq on stamens and carpels undergoing meiosis. Twelve genes were identified as potentially playing a role in pollen viability. *CalS5‐* and *RPG1‐like* genes were identified as pre‐ and post‐meiotic genes for further characterization and to determine their role in pollen viability. It appears that all three homoeologues of both *CalS5* and *RPG1* are functional in wheat as all three homoeologues need to be knocked out in order to cause male sterility. However, one functional homoeologue is sufficient to maintain male fertility in wheat.

**Significance Statement**

We report the identification and characterization of *CalS5‐* and *RPG1‐like* genes as pre‐ and post‐meiotic candidate genes for a genic hybrid system in wheat. CRISPR/Cas9 editing of all six alleles for each gene in several lines results in aberrant pollen morphology and infertility—however, the presence of one allele of Ta*CalS5* or Ta*RPG1* is sufficient for fertility, as plants with only one non‐mutated allele were fertile.

## 1 INTRODUCTION

Wheat is grown on more land than any other crop in the world, with an estimated 240 million hectares grown in 2017 (FAO, 2017). Wheat production is second in the world, after maize, in terms of total tonnage created with nearly 750 million tons produced in 2016 (FAO, 2017). It has been estimated that to meet future demand of an ever‐increasing human population, yields will have to increase sixty percent by 2050 (Tilman, Balzer, Hill, & Befort, 2011). However, yield gains in wheat over the past few years have lagged behind other major crops such as rice and maize (Ray, Mueller, West, & Foley, 2013). One of the main reasons suggested for the lag in yield gains is the lack of a robust hybrid system to harness the potential yield gains associated with heterosis, also known as hybrid vigor. A second potential reason is the lack of focused wheat breeding resources compared with hybrid maize (Whitford et al., 2013). Hybrid systems are thought to offer two advantages: The first is heterotic yield, with gains of over 10% in many crops species easily obtainable (Longin et al., 2013; Longin & Würschum, 2014; Mühleisen, Piepho, Maurer, Longin, & Reif, 2014), and the second is as a driver for private investment in new and elite germplasm to further drive yield gains in prebreeding. However, due to the complex nature of wheat, with a large genome (17 Gb) and allohexaploid nature, the identification of genes involved in male sterility has been hard to establish. This is mainly due to the fact that the wheat genome is approximately forty times larger than rice (0.430 Gb) and seven times larger than maize (2.5 Gb). The added layer of redundancy that polyploids exhibit has meant that mutant screens have produced very few candidate genes involved in male sterility. Therefore, until the recent release of a reference wheat genome, little progress could be made with single mutations or mutant screens (Chapman et al., 2015; Clavijo et al., 2017; Mayer et al., 2014; Zimin et al., 2017).

Wheat being a species which regularly self‐fertilizes is considered as a cleistogamous pollinator, meaning it fertilizes its own stigma before the flower opens. Overcoming the cleistogamy to force outcrossing requires the modification of either timing of pollen release or a mutation to cause sterility (De Vries, 1971). The main mode of action for male sterility usually involves the disruption of the pollen cell wall. The pollen cell wall consists of a simple inner intine layer which is mainly composed of pectin and cellulose, and an intricate outer exine layer composed of sporopollenin, which is made up of fatty acids, phenylpropanoids, phenolics, and carotenoids. In diploid species such as Arabidopsis and rice, it has been found that many of the genes involved in exine formation are highly conserved and play the same roles in a number of plant species (Gómez, Talle, & Wilson, 2015). The two major components of the exine are callose and fatty acids. Callose, which is synthesized at the plasma membrane by an enzyme called callose synthase 5 (CalS5), plays multiple roles in pollen development, as it is involved in covering microspores as they form independent exine layers, but is also a major constituent of pollen tubes (Taylor & Hepler, 1997). The loss of CalS5 causes male sterility in Arabidopsis due to the degeneration of microspores in the mutant due to the failure of normal development of the bacula and tectum, which make up the sexine portion of the exine. Plant growth and development in *cals5* mutants are otherwise normal, and female fertility is not affected (Dong, Hong, Sivaramakrishnan, Mahfouz, & Verma, 2005). *CalS5* is thought mainly to be a pre‐meiotic gene as sterility from the lack of function is caused before the tetrad is formed (Nishikawa, Zinkl, Swanson, Maruyama, & Preuss, 2005). Other genes known to play a role in exine formation and targets for genic hybrid systems are *DEX1* (Paxson‐Sowders, Dodrill, Owen, & Makaroff, 2001), *NEF1* (Ariizumi et al., 2004), *NPU* (Chang et al., 2012), and *RPG1* (Guan et al., 2008). The *dex1* mutant has reduced and delayed exine formation, and the membrane undulation fails to form in dex1 mutants (Paxson‐Sowders et al., 2001). In the *nef1* mutant, exine appears more coarse than that of the wild type, and nef1 microspore membrane undulation is abnormal (Ariizumi et al., 2004). In *npu* mutant, primexine is completely absent and no microspore plasma membrane undulation is observed (Chang et al., 2012). The *RPG1* gene, a member of the MtN3+/saliva gene family, is important for primexine formation, although its detailed function is not currently clear.

While only one male fertility/sterility candidate gene from a model species has been translated to wheat or other major crops, a few loci in wheat have been identified. The single gene translated from a model species to wheat (*ms1*) was originally found in wheat almost 60 years ago as part of the identification of a number of male‐sterile mutants in wheat (Pugsley & Oram, 1959). Which is part of a suite of stable genic male sterility (GMS) loci (*MS1–MS5*) identified thus far in wheat (Driscoll, 1977; Fossati & Ingold, 1970; Pugsley & Oram, 1959; Sasakuma, Maan, & Williams, 1978; Zhou, Wang, Feng, Ji, & Wang, 2008), the five mutants identified contained *ms1* and *ms5* which are recessive mutants (Klindworth, Williams, & Maan, 2002; Sasakuma et al., 1978), and *Ms2*, *Ms3*, and *Ms4* which are dominant mutants (Maan, Carlson, Williams, & Yang, 1987; Maan & Kianian, 2001; Qi & Gill, 2001). Currently, only one dominant gene, *Ms2*, and one recessive mutant have been cloned in wheat (Ni et al., 2017; Tucker, Baumann, & Kouidri, 2017; Wang et al., 2017; Xia et al., 2017), with *Ms2* mutants being widely used for wheat breeding and potentially for hybrid wheat breeding (Ni et al., 2017). Other genes involved in male sterility in wheat include the genes *Ms26* and *Ms45* which are involved in the formation of the pollen cell wall similar to *ms1* which is a transcription factor and regulates the post‐meiotic development of the anther (Dong et al., 2005; Singh, Kumar, Thilges, Cho, & Cigan, 2017; Tucker et al., 2017; Wang et al., 2017). Here, we set out to identify other genes involved in pollen viability in wheat and show their possible use as genes involved in a genic male sterility system. Two genes in particular, one gene from the literature listed as potentially a pre‐meiotic gene (*TaCalS5*) and one potential post‐meiotic gene (*TaRPG1*), are characterized for their role in wheat pollen development and viability (Dong et al., 2005; Guan et al., 2008).

## 2 MATERIALS AND METHODS

### 2.1 RNASeq

RNASeq was performed on total RNA extracted from stamen and pistil samples isolated from immature flowers at meiosis. Total RNA was extracted from three biologically replicated samples of developing stamens and carpels of wheat (*Triticum aestivum)* cultivar Fielder. Tissues were selected and dissected from wheat ears between the Zadok stages 41 and 49, and total RNA was isolated using Qiagen's RNeasy^®^ Kit. Samples were then treated with DNAse to remove any further genomic contamination and purified using RNeasy MinElute^®^ columns. Six RNASeq libraries (three from stamens and three from pistils) were generated for using Illumina HiSeq 2,500 paired‐end reads. The cDNA libraries were treated with the enzyme Ribo‐Zero (Illumina) to reduce the abundance of ribosomal RNAs before the libraries were run on the Illumina HiSeq2500. Sequencing was performed by Eurofins Genomics.

Reads obtained from the six libraries were analyzed using bioinformatics software tool “fastQC” to identify adapter contamination. Adapter contamination was removed from the reads using “cutadapt” software, and trimmed sequences were again run through fastQC to assure adapters had been removed. Trimmed reads were aligned to the TGAC cDNAs using Salmon (Patro, Duggal, Love, Irizarry, & Kingsford, 2017), and reads were quantified using EdgeR after removal of and cDNAs which had less than 10 total mapped reads total across the six libraries (McCarthy, Chen, & Smyth, 2012). A maximum FDR of 0.05 was used as a test of significance.

Validation of the RNASeq results was measured from two more biological replicated the same as above. cDNA synthesis was conducted on 500 ng of total RNA using Omniscript RT Kit (Qiagen). The cDNA was diluted 1:2 with water, and 0.5 µl was used as template in each RT‐PCR. Transcripts levels were quantified using SYBR Green JumpStartTaq ReadyMix (SIGMA) with the standard run conditions for the ABI 7900 HT. Four technical replicates were performed on each of the biological replicates. Two reference genes *TaUbiquitin* and *TaEF1α* were used for the normalization. The sequence of primers used in QPCR assays is shown in Table S9.

### 2.2 Bioinformatic comparison

Putative gene targets were identified from the RNASeq dataset, and identifiers were found in ENSEMBL plants (http://plants.ensembl.org/index.html) and the BioMart option of the site was used to identify conserved PFam domains listed under the transcript portion of a fertility candidate. The populated list of putative genes with the family‐specific PFam domain was then exported as either a fasta format to compare sequences information or .xls format for other data manipulation of the dataset. Splice variants of the same unique identifier in the wheat genome were then removed so only one variant was used for comparison. In most cases, if the gene had no prior characterization, the first splice variant was used for simplicity in the comparison.

RNA‐seq data have been deposited in the ArrayExpress database at EMBL‐EBI (www.ebi.ac.uk/arrayexpress) under accession number E‐MTAB‐8675.

### 2.3 CRISPR‐mediated knockout

To produce plants with targeted mutations in *TaRPG1* and *TaCalS5‐like* genes, we used a CRISPR Cas9 system with a wheat codon‐optimized *Cas9* to introduce mutations in wheat plants (Howells, Craze, Bowden, & Wallington, 2018). We targeted *TaRPG1‐* and *TaCalS5‐like* genes with four guide RNAs for each set of homoeologues. To identify the target sequences in these genes, we used the publicly available program DREG (http://emboss.sourceforge.net/apps/cvs/emboss/apps/dreg.html) to find sequences that match either 5’‐A(N)_20_GG‐3’ or 5’‐G(N)_20_GG‐3’ in both orientations of the Chinese Spring genomic sequence. We then selected four guides based on the following criteria: that the target sequence was conserved in all three homoeologues, that it was (at least partially) in an exon of *TaRPG1‐* and *TaCalS5‐like genes*, and that it had a restriction enzyme site near the site of the protospacer associated motif (PAM) but in the sequence of the guide RNA and prioritized guides near the start of the coding sequences of each gene. The guide sequences selected are shown in Table S10. For targeting *TaCalS5,* one guide was driven by the *OsU3, OsU6, TaU3,* and *TaU6* promoters with a total of four guides targeting *TaCalS5* genes. For targeting the *TaRPG1‐like* gene, we duplicated the TaU6 promoter as we were unable to find a sequence in the *TaRPG1* gene that could fulfill all our criteria for quality guides. These two promoter‐guide constructs were then synthesized (GenScript) and subsequently cloned into an intermediate vector containing attL1 attR5 flanking sites prior to a 2‐way MultiSite Gateway recombination with a wheat codon‐optimized *cas9* sequence driven by the maize ubiquitin promoter flanked by attL5 and attL2 sites, into the final binary vector (Figures S4 and S5). Completed constructs were verified by restriction digest and sequencing before being electro‐transformed into *Agrobacterium tumefaciens.* Plasmids were re‐isolated from Agrobacterium cultures and verified by restriction digest prior to use in wheat experiments (Bates, Craze, & Wallington, 2017).

#### 2.3.1 Wheat transformation

Wheat variety Fielder plants were grown in controlled environment chambers (Conviron) at 20°C day/15°C night with a 16‐hr day photoperiod (approximately 400 μE m^−2^ s^−1^). Immature seeds were harvested for transformation experiments at 14–20 days post‐anthesis (dpa). Isolated immature wheat embryos were co‐cultivated with *Agrobacterium tumefaciens* for 2 days in the dark (Ishida, Tsunashima, Hiei, & Komari, 2015).

Subsequent removal of the embryonic axis and tissue culture was performed as described by Risacher, Craze, Bowden, Paul, and Barsby (2009). Individual plantlets were hardened off following transfer to Jiffy‐7 pellets (LBS Horticulture), potted up into 9‐cm plant pots containing M2 and grown on to maturity, and seed harvest in controlled environment chambers, as above.

#### 2.3.2 DNA analysis of transformed wheat plants

Plantlets which regenerated under G418 selection in tissue culture and transferred to Jiffy‐7 pellets were validated using an *nptII* copy number assay relative to a single‐copy wheat gene amplicon, GaMyb, normalized to a known single‐copy wheat line (Milner et al., 2018). Plants were then screened for mutations using a PCR‐based method where the PCR products were designed to be homoeologue‐specific and covering all four guides or all three homoeologues containing a pair of guides.

### 2.4 Plant rescue of male sterility after CRISPR knockouts

Plants which appeared sterile were then fertilized with Wt Fielder pollen to rescue the male sterility and generate T_1_ seed. All sterile plants had two ears left unfertilized to check male sterility was not tiller specific.

## 3 RESULTS

### 3.1 RNASeq to identify pollen‐specific expressed genes

To identify candidate genes for use in a genic male sterility system, a differential expression analysis was performed on immature stamen and pistil tissues from wheat (cv. Fielder) inflorescences harvested between Zadok stages 41 and 49 (the “booting” stage of wheat development when meiosis takes place). Reads were mapped to the current wheat transcriptome (ref_seq v1.1). From this analysis, a total of 19,490 genes were found to be differentially expressed between the two tissues. A total of 10,474 were more highly expressed in stamens (Table S1), whereas 9,016 genes were more highly expressed in pistils (Table S2). GO analysis did not reveal informative GO terms associated with gene expressed in the stamens compared with pistil tissues (Tables S3 and S4).

Ten genes, which could be inferred to known male‐sterile phenotypes in other plant species or were members of the Sugars Will Eventually be Exported Transporter (*SWEET*) family and highly expressed in the stamens, were prioritized as potential stamen‐specific candidates for knockdown by RNAi silencing. Clear orthologs could be found in the genome sequence publicly available at that time (2015), and three homoeologues were easily identified (Table 1). Five potential candidates, which met these criteria and which showed differential expression based on the RNASeq data, were further confirmed by qRT‐PCR with two new biological samples. All five of the genes chosen were confirmed to be differentially expressed using qRT‐PCR although some of the values were greater or less than measured by RNASeq (Figure S1).

**Table 1:** Candidate genes which could be used in a genic male sterility hybrid system identified from differential expression of developing wheat stamens and carpels

Potential candidate	BLAST hit	TGAC v1 gene model	TGAC v1 homeologues	Significant homoeologues
1	RPG1 (Ruptured Pollen Grain1) like	TRIAE_CS42_7DL_TGACv1_603435_AA1983700	TRIAE_CS42_7AL_TGACv1_556969_AA1774370; TRIAE_CS42_7BL_TGACv1_580455_AA1914070	ABD
2	Callose synthase 5	TRIAE_CS42_7BS_TGACv1_593715_AA1953990	TRIAE_CS42_7AS_TGACv1_569258_AA1811650; TRIAE_CS42_7DS_TGACv1_622598_AA2042310	ABD
3	Aborted microspore 1 like	TRIAE_CS42_6AS_TGACv1_486918_AA1566480	TRIAE_CS42_6BS_TGACv1_514404_AA1659330; TRIAE_CS42_U_TGACv1_643846_AA2135420	ABD
4	RPG1 (Ruptured Pollen Grain1) like	TRIAE_CS42_5BS_TGACv1_423307_AA1373980;	TRIAE_CS42_5AS_TGACv1_393366_AA1271880; TRIAE_CS42_5DS_TGACv1_457788_AA1489840	AB
5	bHLH91	TRIAE_CS42_2AL_TGACv1_094707_AA0301850	TRIAE_CS42_2BL_TGACv1_129925_AA0399500; TRIAE_CS42_2DL_TGACv1_158620_AA0523420	ABD
6	GAMYB (AtMYB101)	TRIAE_CS42_6AS_TGACv1_485682_AA1550030	TRIAE_CS42_6DS_TGACv1_543879_AA1744870	AD
7	Hothead	TRIAE_CS42_4BL_TGACv1_320326_AA1035360	TRIAE_CS42_4DL_TGACv1_343496_AA1135340; TRIAE_CS42_5AL_TGACv1_375593_AA1224180	ABD
8	Hothead	TRIAE_CS42_6DL_TGACv1_527115_AA1698830	TRIAE_CS42_6AL_TGACv1_470984_AA1500160; TRIAE_CS42_6BL_TGACv1_500863_AA1610910	ABD
9	Member of the sweet family	TRIAE_CS42_2DS_TGACv1_177708_AA0582810	TRIAE_CS42_2AS_TGACv1_113352_AA0354890; TRIAE_CS42_2BS_TGACv1_149844_AA0497680	ABD
10	member of the sweet family	TRIAE_CS42_7AS_TGACv1_570345_AA1834200	TRIAE_CS42_7BS_TGACv1_591914_AA1925470	A
11	Similar to OsSweet7e	TRIAE_CS42_U_TGACv1_640821_AA2075730	no strong hit	U
12	Sweet4	TRIAE_CS42_1DL_TGACv1_065128_AA0236610	TRIAE_CS42_1AL_TGACv1_002319_AA0040790; TRIAE_CS42_1BL_TGACv1_030610_AA0095680	ABD
13	Hothead	TRIAE_CS42_1DL_TGACv1_063432_AA0227210	TRIAE_CS42_1AL_TGACv1_001690_AA0034080; TRIAE_CS42_1BL_TGACv1_032570_AA0131570	ABD
14	Ms26	TRIAE_CS42_4AS_TGACv1_308399_AA1027760.1	TRIAE_CS42_4BL_TGACv1_321123_AA1055760; TRIAE_CS42_4DL_TGACv1_345634_AA1154040	ABD

Note

NB Most of the above genes are shown in Table 2 of patent filing PCT/US2017/043009; they are designated *Mfw* genes cross‐referenced to the above Blast Hits, etc. A FDR cutoff of 0.05 was used for significance. Shown are the TGAC v1 representative gene models along with putative homeologues and whether the homoeologue(s) were also significantly differentially expressed.

Two candidate genes were selected based on their characterization in other plant species as being either pre‐ or post‐meiotic developmentally sterile, and one candidate from each category was chosen for further study. Each gene had three clear homoeologues identified, plus significantly different expression in stamen and pistil tissues from the RNASeq dataset, which was also confirmed by qRT‐PCR analysis. The two candidates chosen for further study were a Ruptured Pollen Grain‐like (*RPG1*) and a Callose Synthase‐like (*CalS5*) gene which were selected for further analysis and to determine whether manipulation of these genes would result in male‐sterile wheat.

### 3.2 Bioinformatic analysis of *TaCaLS5* and *TaRPG1*

To understand the conservation of the two candidate genes, public databases were used to ensure that the proper targets were identified. A conserved domain for each protein which helped to define the family was chosen to help identify all putative members of the family in wheat. For *CalS5,* searching for the PFam domain PF14288 in wheat returns 13 loci on the A genome, 14 loci on the B genome, 10 loci on the D genome, and one unanchored locus. A BLAST search of public EST databases showed that only one locus on A, B, and D genomes is expressed only in the anther or pollen and was likely to be homoeologous group loci. These loci TraesCS7A02G146200, TraesCS7B02G048700, and TraesCS7D02G147700 under current refseq v1.1 locations were all differentially expressed in our RNASeq dataset and thus would be the target of future characterization. The overall number of unique loci in the *CalS* family in wheat is similar to that of Arabidopsis which has 12 unique loci. A phylogenetic comparison of the 41 loci in wheat, listed in Table S5, relative to the *AtCalS5* and *OsCalS5* is shown in Figure S2.

The *RPG1‐like* genes which are members of the *SWEET* family of genes showed 108 loci in wheat with the PFam domain PF03083. Again there was an uneven distribution in the genome. There are 29 loci on the A genome, 39 on the B genome, 30 on the D genome, and 10 unanchored. Unlike the *CalS5‐like* candidates, there was not a clear *RPG1‐like* candidate as many different SWEET transporters were expressed in the pollen or stamens. Table S1 shows that there are different members of the SWEET family which are differentially expressed residing on chromosomes 1, 2, 3, 5, and 7. The three potential homoeologues on chromosome 7 were chosen for further analysis as these three were the most highly expressed of the potential candidate genes in a previous iteration of the genome. Although with current genome sequence a triplicate set of homoeologues can also be found on wheat chromosomes 1 and 2, for which the genes on chromosome 2 did match our cutoff (Table 1). However, since the 112 loci are more than three times the 17 members of the family found in Arabidopsis, redundancy and potentially miss‐assignment of orthologous genes may occur. A phylogenetic comparison of the 112 loci in wheat, listed in Table S6, relative to the AtRPG1 is shown in Figure S3.

### 3.3 CRISPR‐mediated knockout of *TaRPG1* and *TaCalS5*

A construct was designed to reduce expression of the *TaCalS5* and *TaRPG1* genes simultaneously through RNAi silencing, with the potential to cause male sterility in wheat. Thirty‐nine positive transgenic plants were generated, of which two showed male sterility, with unfilled and collapsed pollen (data not shown). The two male‐sterile plants set seed only after crossing with wild‐type (Wt) pollen or pollen from another RNAi line.

**Figure 1:**
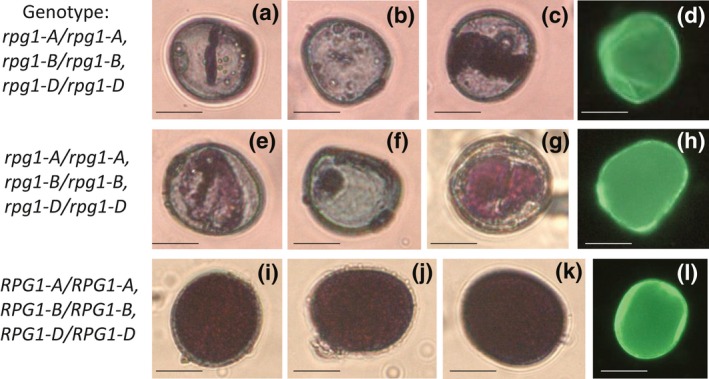
Disrupted pollen formation in *TaRPG1*. Mature pollen stained with either Alexander stain (a‐c, e‐g, i‐k) or Auramine‐O (d, h, l) from male‐sterile CRISPR lines RPG 12 with the genotype *rpg1‐A/rpg1‐A, rpg1‐B/rpg1‐B, rpg1‐D/rpg1‐D* (a‐d), and RPG 13 with the genotype *rpg1‐A/rpg1‐A, rpg1‐B/rpg1‐B, rpg1‐D/rpg1‐D* (e‐h), and Wt Fielder (i‐l) bar = 50 µm

**Figure 2:**
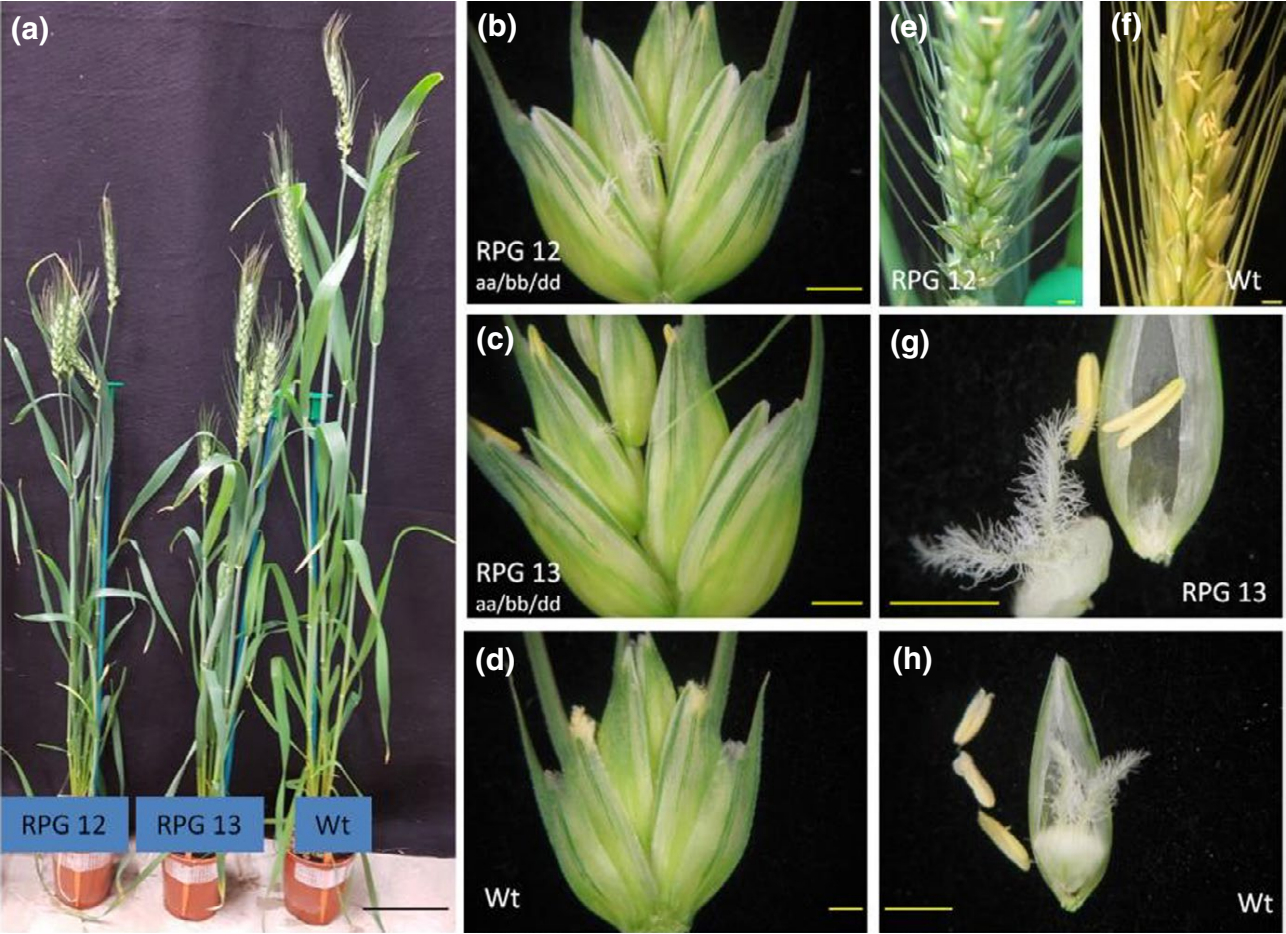
Plant growth and female fertility of *TaRPG1* knockout lines 12 and 13. Images of whole plants (a), florets (b‐d), an ear (e‐f), and dissected flower (g‐h). Bar in A = 10cm, B‐H = 1mm

**Figure 3:**
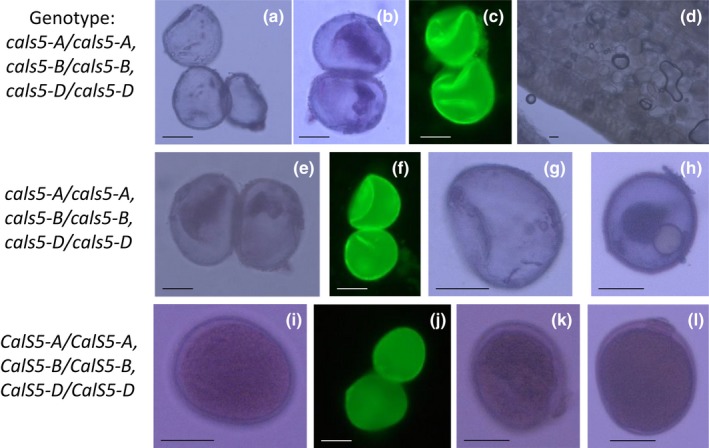
Disrupted pollen formation in *TaCalS5*. Mature pollen stained with either Alexander stain (a, b, d, e, g, h, i, k, and l) or Auramine‐O (c, f, j) from male‐sterile CRISPR lines CalS 5 with the genotype *cals5‐A/cals5‐A, cals5‐B/cals5‐B, cals5‐D/cals5‐D*, (a‐d), and CalS 17 with the genotype *cals5‐A/cals5‐A, cals5‐B/cals5‐B, cals5‐D/cals5‐D*, (e‐h), and Wt Fielder (i‐l) bar = 50 µm

**Figure 4:**
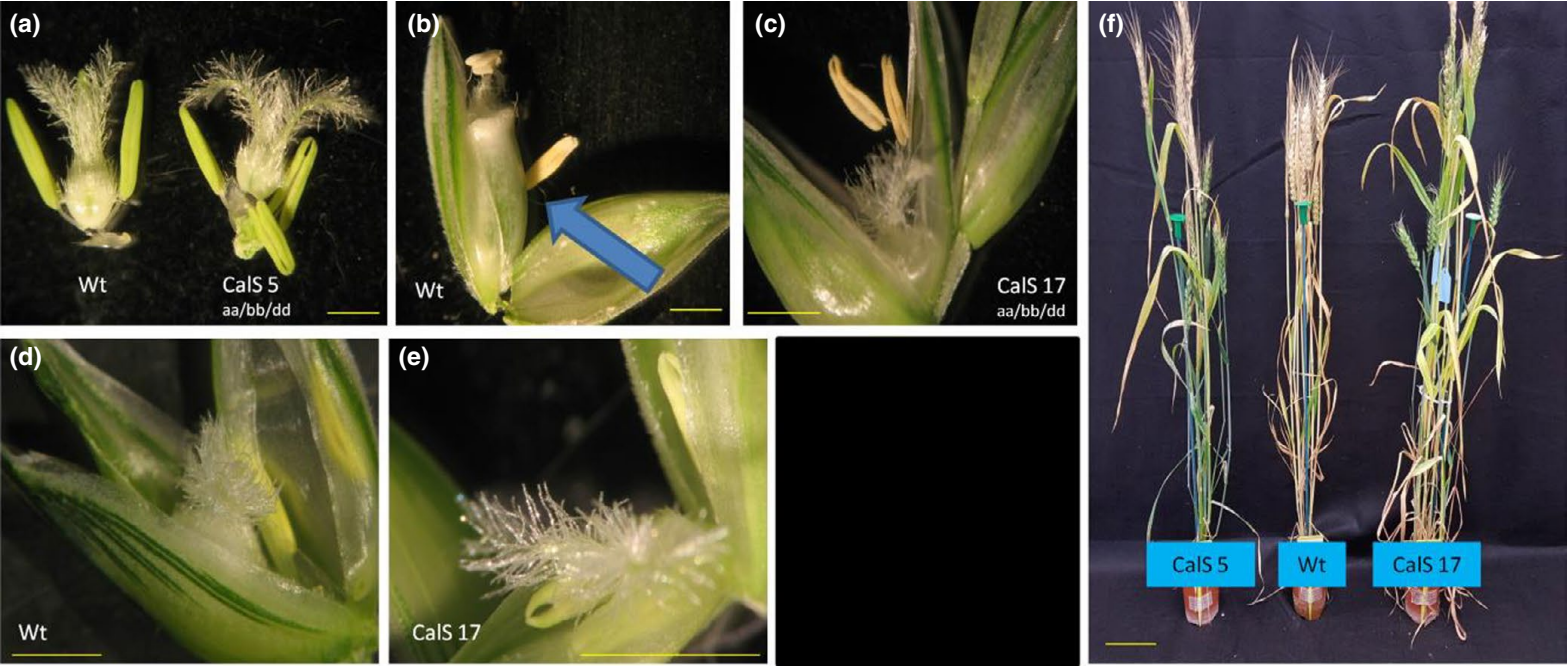
Plant growth and female fertility of *TaCalS5* knockout lines 5 and 17. Images of dissected flower before fertilization (a) after fertilization could occur (b‐c), florets (d‐e), and the entire plant (f). Bar in a‐e = 1 mm, *f* = 10 cm

To understand which, or both, pollen expressed genes were causing the sterility phenotype, four guides were designed to target all three homoeologues of either *TaRPG1* or *TaCalS5* (Figures S4 and S5). Wheat transformation experiments with two CRISPR/Cas9 constructs generated a total of 64 T_0_ plants with guides targeting *TaRPG1* and 101 plants targeting *TaCalS5*. Nine of the 64 *TaRPG1*‐targeted plants (14%) were sterile, and 7 of the 101 *TaCalS5‐*targeted plants (7%) were sterile. Pollen from sterile plants was analyzed and showed the same phenotype as the RNAi lines, with unfilled and collapsed pollen grains (Figures 1, 2, 3, 4). All sterile plants from each of the CRISPR‐targeted genes were fertilized with Wt Fielder donor pollen to ensure sterility was indeed male specific. All sterile plants (9 *TaRPG1* sterile plants and 7 *TaCalS5* sterile plants) were rescued by Wt donor pollen, confirming the male‐sterile phenotype, and generating T_1_ seed for further analysis. No observable differences were detected in transformed plants compared with untransformed control Wt Fielder lines, except for the male sterility. The obvious phenotype of the male‐sterile plants was gaping phenotype of the flowers after anthesis (Figure 2b,c, and e) This suggests no deleterious effects from the loss of either of these genes on normal growth patterning or any other agronomic trait of interest (Figures 1, 2, 3, 4).

### 3.4 Identification of CRISPR‐mediated mutations in *TaRPG1* and *TaCalS5*

To understand the basis of the sterility, the homoeologous regions targeted by the guides were sequenced in 20 plants per construct including both sterile and fertile plants. For guides targeting *TaRPG1*, two plants contained no mutations, 8 plants contained a range of mutations on either one or two of the homoeologues targeted, and two plants each contained a full knockout of all 6 alleles (Table S7). No plant with less than a mutation in all six *RPG1* alleles showed a male‐sterile phenotype. All of the predicted changes to the coding sequences from Table S7 are shown in Figures [Link], [Link], [Link].

For *TaCalS5*‐targeted plants, only one plant contained no mutations at any of the four guide target sites sequenced. One plant (CalS 3) contained six different mutations but as one allele on the B genome contained a 3 bp mutation, resulting in 2 amino acids being substituted by one amino acid, with the rest of the protein conserved in frame, fertility was maintained. The range of deletions ranged from a deletion of 1,029 bases to the addition of 118 bases on one allele (Table S8). *TaCalS5‐*targeted plants required all six alleles to be mutated to cause male sterility; seven plants had a complete knockout of all six alleles and exhibited the resultant sterile phenotype. Again all of the predicted changes as a result of the mutations are shown in Table S8 and Figures [Link], [Link], [Link]. It should also be noted that neither of the OsU3 guides produced any mutations for either gene targeted. Of the male‐sterile plants identified for both *TaRPG1* and *TaCalS5* genes, every sterile plant had mutations in all six alleles. Among the fully fertile plants, two had only one Wt allele (with the other five being mutated): 1 *TaRPG1* plant (RPG 11) and 1 *TaCalS5* plant (CalS 3).

## 4 DISCUSSION

With an ever‐improving reference genome, identification of wheat orthologues involved in pollen development can now occur at greater speeds. This, along with the development of ever‐increasing public resources such as public expression databases and publicly available TILLING mutants, has led to a new dawn in the ability to finally translate some of the gains from other species such as Arabidopsis and rice to more complex genomes such as wheat. The release of the wheat genome data along with the rapid development of gene‐editing/genomic modification tools such as CRISPR‐Cas9 has resulted in a major step forward in the development of a hybrid wheat breeding system.

Here, we set out to identify genes which might be involved in pollen viability and show two such examples of how this can be applied to genic hybrid systems. From the original RNASeq dataset, we were able to identify fourteen candidate homologous gene sets as potentially being vital in the pollen fertility. From this list, two genes involved in cell wall formation were further characterized for their potential effects on creating sterility in wheat. As shown by CRISPR‐mediated knockout, both genes studied here are involved in maintaining the integrity of the pollen cell wall, while the bioinformatic comparison of the two genes characterized summarizes the potential transfer of knowledge from other plants to wheat. For example in the case of *TaCalS5* there was a clear orthologous set of genes on chromosome 7; this along with the expression data from public databases clearly shows only one clear candidate showing about 77% homology to the Arabidopsis orthologue. This even carries through to the rough estimation of family size, as 12 genes were found in diploid Arabidopsis and 38 were found in hexaploid wheat. However, in the case of *TaRPG1,* the direct transfer of this knowledge was not straightforward as it was found that at least four separate homoeologue sets could potentially be involved in pollen development.

We identified 21 such members of the SWEET family of transporters which were differentially expressed in stamens versus pistils including homoeologous groups on chromosomes 1, 2, and 7. This is not surprising as in Arabidopsis at least three members of the family have already been shown to be important in pollen development or viability (*AtSweet5, AtSweet8,* and *AtSweet13*) (Engel, Holmes‐Davis, & Mccormick, 2005; Guan et al., 2008; Sun, Huang, Yang, Guan, & Yang, 2013). While we targeted the *TaRPG1/Sweet8* member in our work presented here, there appears to be additional potential targets as genes for manipulation in a genic hybrid system. However, as our 21 loci were more highly expressed in stamen tissues, it would suggest that, at least in wheat, there may be more sugar transporters involved in pollen formation than in Arabidopsis and therefore greater potential for redundancy.

**Figure 5:**
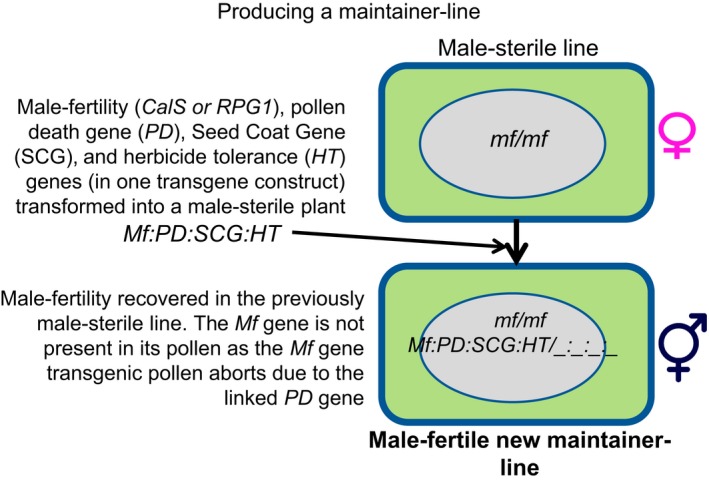
Scheme to produce a maintainer line using a genic male sterility gene to force outcrossing. Figure adapted from Wan et al., 2019. The system would also use other marker genes to prevent the release of the functional copy of the male sterility gene, and the selection for a heterozygote maintainer

RNAi‐silencing, as an established technology, was used at the first stage to provide evidence of the genes involved in male fertility in wheat. However, its use in a commercial wheat hybrid system is of limited interest due to it having a “dominance” effect: The expression of one exogenous RNAi allele can silence all Wt alleles. In contrast, gene knockout technology, such as CRISPR‐Cas, creates mutant alleles which are usually recessive to wild‐type alleles—preferably to such an extent that just one Wt allele will suffice for expression. This has major attractions for a hybrid wheat system as an elite line (wild‐type male fertility) pollinator may be used to produce a fertile hybrid. This is why, when it became available, CRISPR‐mediated mutation was used in the second phase of our work. A workable model has been proposed by Wan et al. (2019) and has been slightly modified for this work highlighting the role of a genic male‐sterile system in wheat (Figure 5).

Using CRISPR/Cas9 editing, we were fortunate in our ability to show a direct phenotype in the T_0_ gene‐edited plants; more work needs to be done to determine which homoeologue is the best candidate for a genic hybrid system with these two genes. Our data suggest that in contrast to *Ms26* in wheat, one functional allele appears sufficient to allow normal seed set (Singh et al., 2017). We also show the power of combining multiple guides in one gene‐editing cassette as we achieved an overall success rate of knocking out all six alleles in 14% of the T_0_ plants in the case of *TaRPG1*‐targeted homoeologues. With the advances in guide prediction and a better understanding of promoters driving expression of the guides, this level of success may be increased to that of diploid species such as rice where it is routine to achieve mutations in 90% of the T_0_ plants regenerated (Miao et al., 2013). This can be illustrated in Figure 5 which shows how a genic male sterility system can be maintained using a genic male sterility gene (adapted from Wan et al., 2019).

This dataset can also be used for other future advancements in genic male fertility/sterility hybrid breeding systems as we identified 62 pentatricopeptide repeat‐containing proteins (PPR) which are the basis of a number of cytoplasmic male hybrid breeding systems in rice (Akagi et al., 2004; Dahan & Mireau, 2013; Hu et al., 2012; Kazama & Toriyama, 2003; Zhang, Lu, Bharaj, Virmani, & Huang, 1997). However, since the PPR family of proteins is a poorly conserved and complex family, identifying the direct orthologues of known rice genes is not a simple task (Dahan & Mireau, 2013). In our dataset, there were no clear orthologues of known PPRs which matched known rice *Rf* genes. A thorough understanding of the role played by these mitochondrial proteins and a route for their efficient manipulation for a functioning genic hybrid system to work are areas for future study and investment.

## CONFLICT OF INTEREST

M.M. and A.K. have filed a patent application based on the results reported in this paper. All other authors declare no competing financial interests.

## AUTHOR CONTRIBUTIONS

M.M., E.J.W., and A.K. designed the experiments and wrote the manuscript. M.M. conducted the experiments and analyzed the data. M.C., S.B., and R.B. generated the transgenic plants and assisted with microscopy.
