# Supplementary material for: Identification of genes involved in male sterility in wheat (Triticum aestivum L.) which could be used in a genic hybrid breeding system
Source: Plant Direct. 2020 Mar 10;4(3):e00201. doi: 10.1002/pld3.201 (PMC7063588; doi:10.1002/pld3.201)
Supplement: Supplementary file 1 [file PLD3-4-e00201-s001.docx]

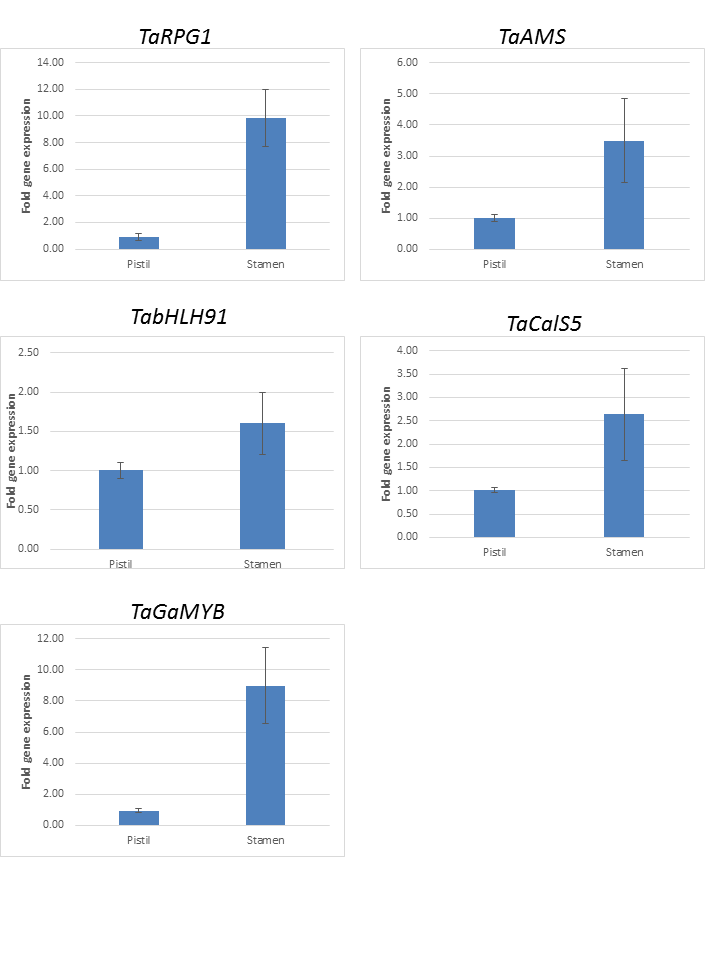


Suppl. Figure 1: qRT-PCR of five candidate genes selected from table one for their expression in stamen or pistil tissues during or around time of meiosis. All gene changes shown are significant with p val <0.05 between the tissues tested.
