# Supplementary material for: Identification of genes involved in male sterility in wheat (Triticum aestivum L.) which could be used in a genic hybrid breeding system
Source: Plant Direct. 2020 Mar 10;4(3):e00201. doi: 10.1002/pld3.201 (PMC7063588; doi:10.1002/pld3.201)

Suppl. Figure 2 Phylogenetic tree of genes which belong to the Callose Synthase family in Wheat taken from the genes in ref seq v1.1


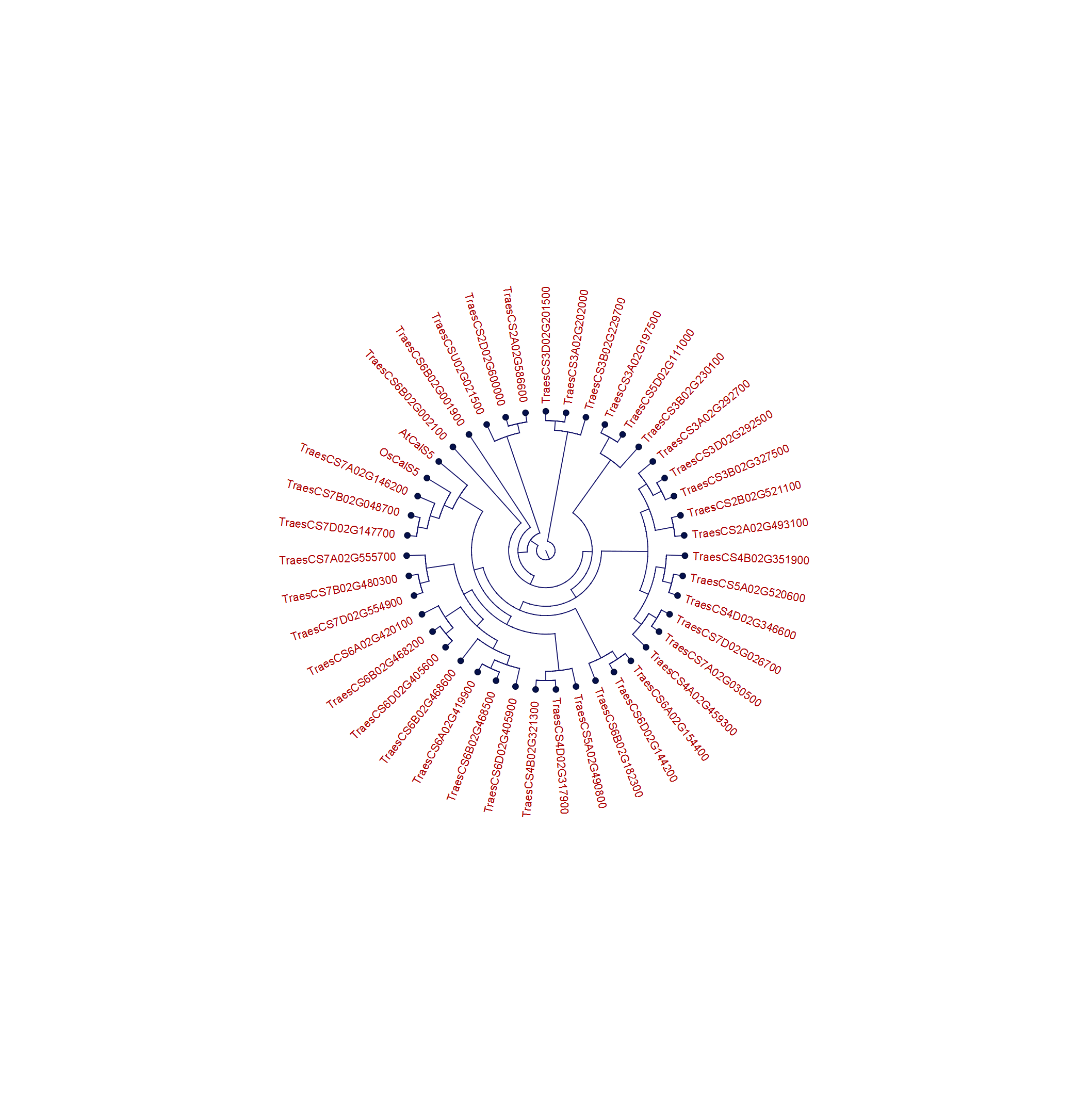

Supplement: Supplementary file 2 [file PLD3-4-e00201-s002.docx]
