# Supplementary material for: Identification of genes involved in male sterility in wheat (Triticum aestivum L.) which could be used in a genic hybrid breeding system
Source: Plant Direct. 2020 Mar 10;4(3):e00201. doi: 10.1002/pld3.201 (PMC7063588; doi:10.1002/pld3.201)
Supplement: Supplementary file 5 [file PLD3-4-e00201-s005.pdf]

A

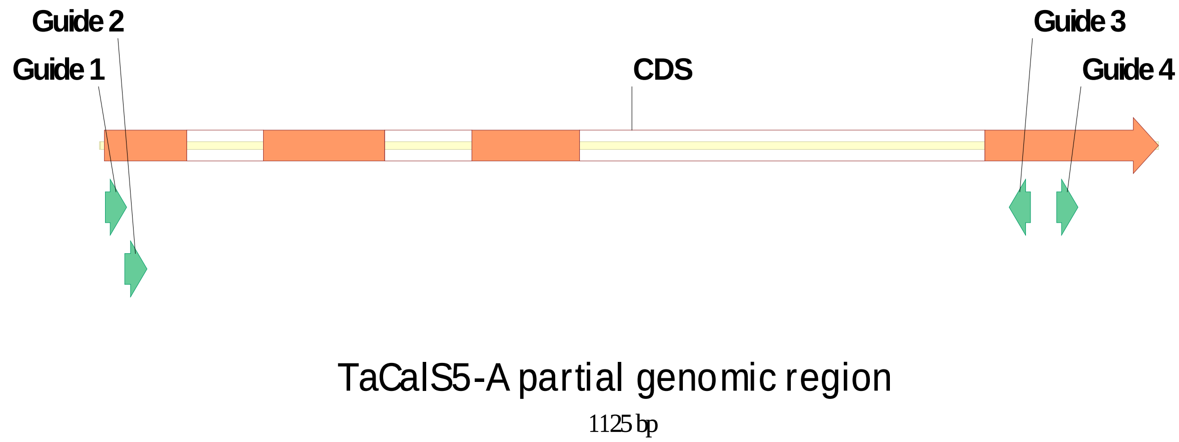

B

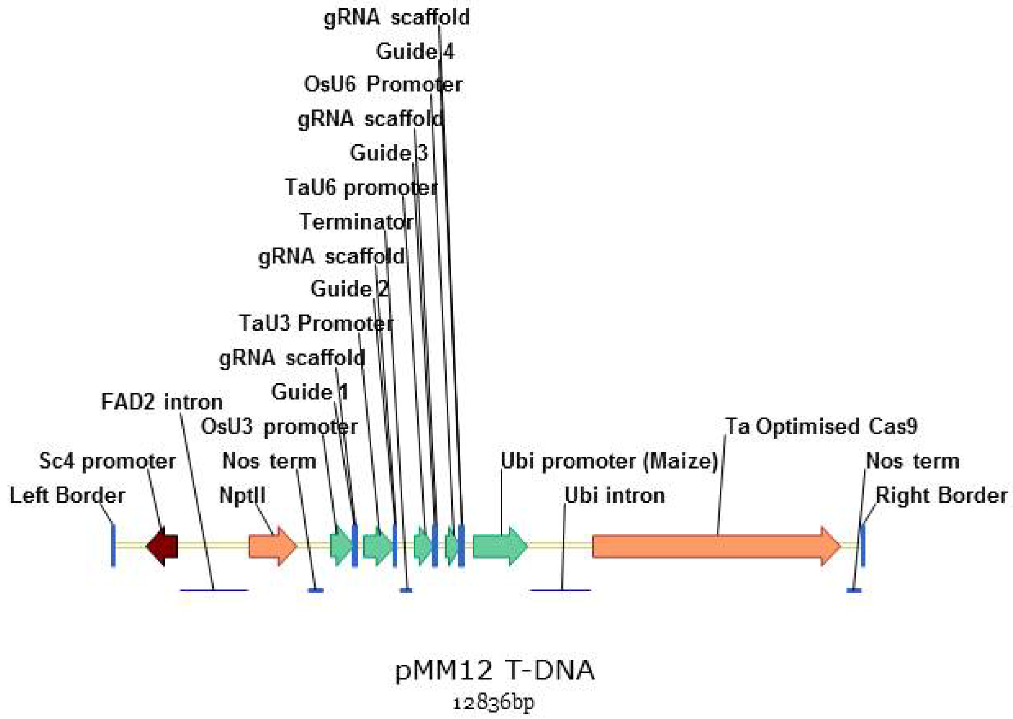

Supplemental Figure 5: *TaCalS5* CRISPR guide location and pMM12 T-DNA structure. A *TaCalS5-A* partial genomic region showing the orientation of the partial CDS covering exons 12-15 (orange blocks and arrow) and location of CRISPR guide target sequences (green arrows). B Schematic of the pMM12 binary plasmid T-DNA region transferred to wheat.
