# Supplementary material for: Identification of genes involved in male sterility in wheat (Triticum aestivum L.) which could be used in a genic hybrid breeding system
Source: Plant Direct. 2020 Mar 10;4(3):e00201. doi: 10.1002/pld3.201 (PMC7063588; doi:10.1002/pld3.201)

Suppl. Figure 6: Predicted amino acid consequences of the CRISPR Cas9 mutations in the *TaRPG1-A* homoeologue of all of the sterile plants produced.

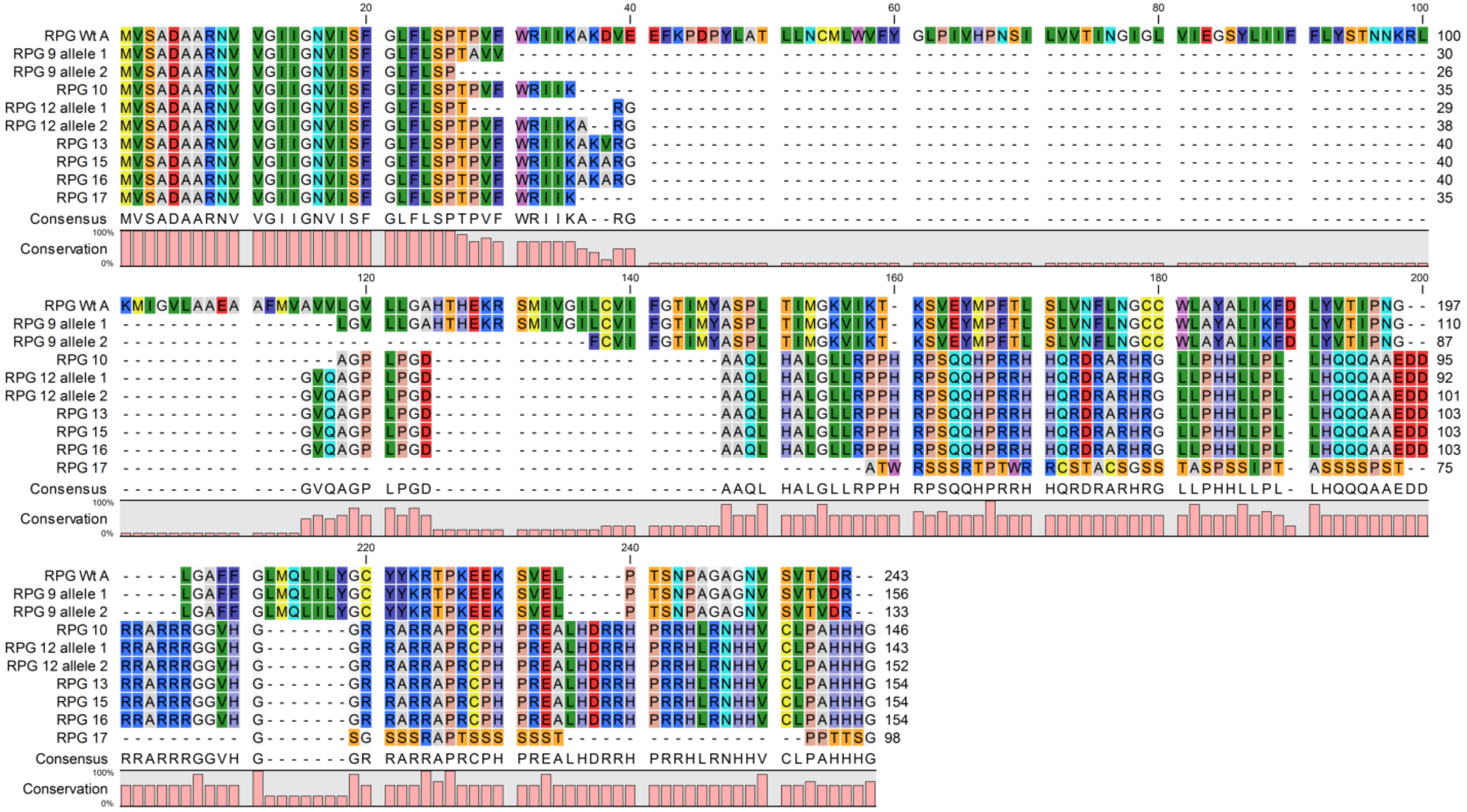

Supplement: Supplementary file 6 [file PLD3-4-e00201-s006.pdf]
