# Supplementary material for: Identification of genes involved in male sterility in wheat (Triticum aestivum L.) which could be used in a genic hybrid breeding system
Source: Plant Direct. 2020 Mar 10;4(3):e00201. doi: 10.1002/pld3.201 (PMC7063588; doi:10.1002/pld3.201)
Supplement: Supplementary file 22 [file PLD3-4-e00201-s022.docx]

**Reviewer 1**

There is no reference to the RNASeq datasets and I am unsure if they will be publicly available, this is essential for future applications of this data.

We will submit the RNASeq dataset to ENA upon acceptance of the m/s. A reference to the ENA identifier is included at the end of the M&M RNASeq section.

Minor points:
Fig legend 1. RNAi include details about target gene in legend Only single line shown? Why not include other independent transformants?

Details of target genes have been added to the legend. We identified two RNAi lines in which expression was sufficiently knocked down to cause sterility, whereas the remaining 37 transgenic lines were insufficiently down-regulated and remained fertile in this preliminary experiment. The second sterile plant was not imaged as we intended to follow this up with detailed CRISPR analysis of the candidate genes.

Fig 2 (& poss 6). I think it would be useful to include genotypes on figures in small text rather than in legend as it is difficult to follow otherwise

Genotype information has been added to figures 2 and 4 as requested

Fig 3 scales needed for florets, ears and flowers

Scale bars and genotypes have been added to Figures 3 and 5 as requested.

**Reviewer 2**

Page 6 L3-49: No table or figure for this section. Authors should generate separated trees for demonstration of various homologues.

Suppl. Figure 2 and 3 show phylogenetic trees of the homeologues listed and are referenced in the Results – Bioinformatics section of the text

Page 7 L2-15: Missing molecular characterization of DNA integration and RNAi occurrence both for male sterile and fertile plants. Missing qRT-PCR verification of down regulation of both genes or just one gene.

Characterization of T-DNA integration in all RNAi lines is included in Supplemental Table 5. Both nptII copy number (QPCR) and RNAi cassette presence data (PCR) is included for each plant. Unfortunately we do not have qRT-PCR data for these plants as it would have been very difficult to collect sufficient tissue for extraction of RNA, whilst also observing the male sterile phenotype and also rescuing the phenotype with donor pollen from a WT plant in the preliminary experiment with the limited number of ears available. If the editor still feels that this qRT-PCR data is required to validate this RNAi experiment sufficiently for publication, we would need to remove this section of the manuscript, as we just do not have the data. This was a preliminary experiment, and we then followed this up with the detailed CRISPR experiments for each of the two candidate genes.

Page 7 L24: Missing a figure for construct map including specific promoter.

The RNAi binary vector and specific promoter (rice Actin) for expression of the hairpin in planta were both referenced in the Materials and Methods section on RNAi silencing. A new supplemental figure 4 has now also been included.

Page 7 L40-42: Need experimental data to conclude "The overall growth of the sterile plants was similar to untransformed control Wt Fielder lines for all parameters except for the male sterility."

We have changed the text of the manuscript to address the reviewers concerns in the previous version of the m/s. The text was changed to “No observable difference were detected between plants other than the male sterility”.

Page 7 L40-42: How about the flower opening? Whether the male-sterile florets remain open waiting for pollen grains? If it is not, how could this gene be used for breeding?

This is the case and as stated in the text on page 8 lines 33-35. As we are sure the reviewer is aware the easiest way to identify male fertility is the gaping of the open flower which does not occur in WT, or when these two genes have not been mutated. This gaping flower waiting for donor pollen from a fertile plant makes crossing easier for a day or two but the fertility of the stigma is also an issue.

Page 7 L1-29: A picture display of sequence variation is more apparent for readers, especially for the eight plants (2 for TaRPG1, 6 for TaCalS5) with null alleles in all six copies

We have created six new supplemental figures showing the predicted changes in the amino acid sequence for each of the three homeologoues for all of the sterile plants produced. We have also added a column to the original Suppl. tables listing their fertility in the last column.

Page 11 L5: With three replicates during Zadok 41-49, how can you tell they are pre-post meiotic genes? How to quantify if there is less than two repeat for each stage?

We have changed the introductory text to highlight that the pre and post meiotic definitions are from other plant species where these genes have been previously characterized

Page 13 L23-25: "Isolated immature wheat embryos were co-cultivated with Agrobacterium tumefaciens for 2 days in the dark (Ishida et al., 2015)." Please clarify the transformation is done as in this paper or using Japan Tobacco's patents. This will help readers to adopt the right method.

We confirm that the co-cultivation stage of the transformation process was carried out as per the Ishida et al., 2015 reference.

Minor comments:

Page 2 L14: "the yield potential associated with heterosis," as well as Page 3 L22.

We have changed the text to be clear and drive the point that “potential” yield increases come both from investment and releasing genetic gains.

Page 2 L28: Change "Our data also suggests that only one homoeologue is necessary for fertility as plants with only one non mutated allele were fertile." TO "However, one functional homoeologue is sufficient to maintain male fertility in wheat."

The abstract has been changed to the text suggested by the reviewer.

Page 3 L9-26: Very similar to abstract, either one of them should be rewritten.

We have rewritten the introduction to further highlight some of the points made in the abstract without being so repetitive.

Page 3 L10: "www.fao.com, 2017" is not a regular reference. It should be replaced.

We have changed the reference from www.fao.com to FAO,2017 as requested

Page 3 L23-24: Grammar problem between sentences. As well as L29.

We have changed the wording of these sentences to improve grammar and clarity..

Page 3 L49: Is it true? "Wheat is a cleistogamous pollinator, meaning it fertilizes its own stigma before the flower opens."

Yes this is true - wheat is a cleistogamous pollinator, as cited in the text this is a known fact in wheat.

Page 3 L49-54: How a sterility can overcome cleistogamy? Clarify this.

Overcoming the cleistogamy is mentioned in the text as “Overcoming the cleistogamy to force outcrossing requires the modification of either timing of pollen release or a mutation to cause sterility.” The plants which are fertile will continue to outcross to other sterile plants in the field as these plants were not pollinated prior to the flower opening.

Page 4 L19-24: It reads awkward. Rephrase the sentence.

We have reworked this sentence to provide a clearer message.

Page 5 L20-26: whet genotypes should be introduced.

We have added the cv Fielder to the text

Page 5 L32-40: How did you prioritize to focus on these two genes?

We have reworked table 1 to highlight a direct comparison with known male fertility genes in other species as well as some identified from the RNASeq data set. However this table no longer matches the information we had at the time when the decisions were made on which candidates to follow up. As the reviewer is most likely aware not all possibilities of known male sterile genes are listed. We would also like to make the reviewer aware that the genes were chosen during a time of rapid changes in the available sequence and gene models of bread wheat. At the time CalS5 and RPG1 were the largest differentially expressed genes for which we could identify all three homoeolgues. As more information has been made available and as the genome has changed some of the mapped reads to a particular gene no longer map to that gene and now are part of a more complete genome.

Page 5 L40-45: Move them to the end of the paragraph.

We have made the suggested changes as the reviewer requested.

Page 5 L45-53: Long-winded with limited information.

We have rewritten this section.

Figure 1: Legend lacks sufficient information. Better to explain aberrant in detail. Also for Fig. 2-5

The detail requested by the reviewer is included in the text on page 7

Figure 2: Add genotypes and developing stages to the figure directly.

This information has been added to the figure

Figure S1 and S2: What does the arrow head represent for?

Orange arrow indicates orientation of the gene, which is now included in legend

Page 12 L15: Need reference for pACTIR2

We have added the reference Milner et al., 2018

Page 13 L3-7: Don't understand this sentence. A construct map will help.

A diagram of each T-DNA has been included

Reviewer: 3


1, how the two candidate genes were isolated from differential expressed 17,267 genes and why are they?

We have reworked the table to highlight a direct comparison with known male fertility genes in other species as well as some identified from the RNASeq data set. However this table no longer matches the information we had at the time of the when the decisions were made for which candidates to go after. As the reviewer is most likely aware not all possibilities of known male sterile genes are listed. We would also like to make the reviewer aware that the genes were chosen during a time of rapid changes in the available sequence and gene models of bread wheat. At the time CalS and RPG1 were the largest differentially expressed genes for which we could identify all three homoeolgues. As more information has been made available and as the genome has changed some of the mapped reads to a particular gene no longer map to that gene and now are part of a more complete genome. Also since half of the genes on expressed more highly in the pistils rather than the stamen that cut the list by about half.

2, Figure 1 it is not clear why the pollens (A,B,C and E, F, G) from the same RNAi plant is different, and no WT controls corresponding to I to P.

Knockdown is incomplete. WT images are now included in the figure.

3, to show the defect in pollen development (in Figures 1,2 and 4), it should be characterized the nucleus in pollen stained by DAPI.

Our goal was to identify pollen which is sterile/non functional not the full developmental process of pollen development in wheat. While we agree this data might be useful to some readers, it was not the goal of the study and therefore we looked at the cell wall, rather than nuclei.

4, the information included in Figures 3 and 5 is very limited, the authors should provide more information for mutant phenotypes.

We have changed the text of the manuscript slightly to address the reviewers concerns. The text was changed to “No observable differences were detected between plants other than the male sterility”

5, the authors should provided data for the silence of two candidate genes in RNAi lines.

The primary transgenics had a limited number of tillers so we prioritised collection of pollen and rescue with donor pollen. There was very little extra material available for collection to analyse expression of the two gene targets. Now that the plants have been rescued and crossed back to WT Fielder we cannot guarantee that the knockdown will still be sufficient to cause a meaningful phenotype to measure. We have included this initial data to show how we ultimately targeted each gene by CRISPR, to show that mutation of either gene caused sterility. If the reviewers feel that the RNAi expression data is essential, we would be happy to remove the RNAi portions of the paper and just show the CRISPR data and related phenotypes.

6, the authors should provided data for the knockout of two candidate genes in CRISPR lines.

We show the causal mutations for the various mutations observed. But showing each of the sequence traces for 40 different plants for each of the three homoeologues would be a massive unreadable file and not provide the reader with any meaningful knowledge. We do break down the mutations by guide and there effects in the supplementary material. There are also a number of CRISPR papers published which do not show the sequences – e.g in The Plant Journal, Zhang et al., 2017 (<https://doi.org/10.1111/tpj.13599>) did not show the mutations in wheat but rather the consequences. Similarly we have included the consequences of the mutations in this manuscript.

7, the authors should indicate one, two or all three homologs were knockout in the CRISPR lines in Figures.

In the figures of the main text all of the plants shown have three homoeologues knocked out. This is included in the figure legend of each figure. Supplemental files contain the mutations for each guide in each homoeologue as part of supplemental tables 6 and 7.

8, the authors should show the consequences of gene deletion by CRISPR for the two candidate genes (generate a premature termination, a frame shift or other defect after the deletion by CRISPR?).

This data is listed as supplemental figures 7-12.


Specific points are as follows:-

• Unclear what analysis of the genotype/expression was done on the 40 RNAi lines- were they carrying functional RNAi constructs for both (TaCalS5 and TaRPG1) genes? Only 2 out of 40 showed impacts on fertility- why? What was the difference between the lines?

We have added a supplemental table of T-DNA copy number and validation of the RNAi cassette by PCR as Suppl. Table 5, which is referenced in the text.

• The CRISPR mutants- what was the detailed phenotype of these lines? Sections of anthers have not been included along with expression analysis of genes in question.

The authors show that the pollen is the mode of action for the sterility seen in the various transgenic plants and not some other developmental aspect of floral development (ie the female parts) as the reason for the sterility. Also the authors show that the female portions of the plant are also not affected from the loss of either gene studied here as the plants could be crossed to WT pollen or seeds produced. No other obvious phenotypes were observed during the growth of these plants other than the opening of the flowers later during ear development which is highlighted in the text.

• There is a lack of phenotypic data associated with the male sterile changes - this needs to be expanded and should include sections of anthers/pollen for morphological analysis.

As mentioned above images of the pollen clearly show a lack of a full/healthy mature pollen grains being produced in the sterile plants. Cross sections of anthers plus SEM or TEM might reveal the exact mode of action but is outside the goals of this manuscript. Our goal was to identify genes in wheat which could be used to create a sterile plant. We have clearly shown that these two genes are involved in pollen formation and that the loss of function of these genes results in a sterile plant. The exact mode of action could be the subject of future study.

• The RNASeq data provides a valuable resource for studies in this area, however has not been analysed to a sufficient level to currently give it independent value.

We have updated the expression data to the most recent genome/transcript data the community has at this point. We also have validated some of the potential candidate genes from the RNASeq dataset to further confirm our findings. We have performed a GO analysis. Further analysis is outside the scope of this paper but as the data will be made publically available, the community can perform those analyses to make additional scientific gains.

• Page7, L15-23: The EST Blast data would be better presented in table format.

New Suppl. tables have been added to assist the reader identify the gene models which fit the criteria mentioned in the sections highlighted by the reviewer.

• Page9, L 3-6 and suppl table: Including male sterile and male fertile phenotype data in supplemental tables 3 & 4 would be helpful to link phenotypes and genotypes.

We have added sterile/fertile column to the suppl. tables 6 & 7 as suggested assist the reader identify the phenotype linked to the mutation data.

• The staging system and validation of the stages for RNASeq is not fully detailed; there needs to be validation that expression changes are not a consequence of staging differences.

As mentioned above we have validated five genes listed in table 1 from a range of gene families to show how many of the genes might plant a developmental role in pollen development. This qRT-PCR data is now listed in Suppl. Figure 1.

• The title is not reflecting the results in manuscript- the manuscript describes genes impacting upon pollen development but is a long way from a hybrid breeding system- reducing the emphasis on this part would be appropriate.

We state that the genes identified here “could” be used in a hybrid breeding system. The first step for creating a hybrid wheat would require male sterility for which we show in the manuscript. To further support our choice of a title we would also point to the recent publication by Tucker et al. Nat Commun. 2017; 8: 869 which use similar language without showing any part of a hybrid breeding system. However we have also added a supplemental figure to the text to show the potential for a genic male sterility hybrid system adapted from Wan et al., 2019 to highlight the potential of male sterility genes to the reader.


Editor comments:
I suggest the use of more informative titles in the sections of results.

Section titles have been expanded as requested

Please clarify whether the RNAseq data has been submitted to public repositories.

As stated above as soon as the m/s is accepted all of the data will be uploaded to ENA

It is important that the authors provide data for the silencing of two candidate genes in RNAi lines and for the knockout of two candidate genes in CRISPR lines.

The primary transgenics had a limited number of tillers so we prioritised collection of pollen and rescue with donor pollen. There was very little extra material available for collection to analyse expression of the two gene targets. Now that the plants have been rescued and crossed back to WT Fielder we cannot guarantee that the knockdown will still be sufficient to cause a meaningful phenotype to measure. We have included this initial data to show how we ultimately targeted each gene by CRISPR, to show that mutation of either gene caused sterility. If the reviewers feel that the RNAi expression data is essential, we would be happy to remove the RNAi portions of the paper and just show the CRISPR data and related phenotypes.

Mutation data plus the fertile/sterile assessment is included in this manuscript for the all of the CRISPR lines, plus images from selected plants.
